# On artificial crystal structure generation for solving the phase problem with deep learning

**DOI:** 10.1107/S2053273325009428

**Published:** 2026-01-01

**Authors:** Džonatans Miks Melgalvis, Toms Rekis

**Affiliations:** a Faculty of Medicine and Life Sciences, University of Latvia, Jelgavas iela 1, Riga LV1004, Latvia; b Institute for Inorganic and Analytical Chemistry Goethe-Universität Frankfurt am Main Max-von-Laue Straße 7 Frankfurt am Main 60438 Germany; Ernst Ruska-Centre for Microscopy and Spectroscopy with Electrons, Forschungszentrum Jülich, Jülich, 52428, Germany

**Keywords:** phase problem, deep learning, artificial crystal structures

## Abstract

We discuss and present approaches for generating artificial crystal structures for training neural networks to solve the phase problem. Structure generation is considered as a two-step process involving sampling unit-cell parameters and filling the unit cell with atoms. The former step includes generating lattice basis vectors from randomly sampled unit-cell volume. Apart from randomly placing atoms, we use database data to guide fast and scalable generation of molecule-like fragments. The recently developed neural network PhAI is then used as a benchmark and retrained with various sets of training data to assess how the corresponding models perform on experimental crystal structure data. We found a significant improvement in PhAI retrained on a new kind of artificial data to generalize the phase problem solution for larger unit-cell structures.

## Abstract 2

A new approach for generating training data for solving the phase problem with deep learning is proposed. Models trained on such data are better at generalizing the solution for structures outside the training domain.

## 1. Introduction

Deep learning is increasingly being used to tackle different scientific questions in crystallography, for example, aiding symmetry determination (Tiong *et al.*, 2020 ▸; Corriero *et al.*, 2023 ▸; Suzuki *et al.*, 2020 ▸; Park *et al.*, 2017 ▸) or indexing of powder patterns (Chitturi *et al.*, 2021 ▸), as well as solving the phase problem (Larsen *et al.*, 2024 ▸; Pan *et al.*, 2023 ▸). Such deep learning approaches rely on using large amounts of data to train neural networks for specific applications. However, it is not always possible to obtain enough experimental data to serve as a training basis and thus artificial data are necessary. The generation of such artificial data should be carefully designed so that the created training domain is a good representation of the real experimental data (Jordon *et al.*, 2022 ▸; Nikolenko, 2021 ▸).

Recently, a neural network called PhAI was developed which is able to solve the crystallographic phase problem for small unit-cell structures in the 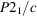 space group and its supergroups (Larsen *et al.*, 2024 ▸). PhAI was trained on 48 million artificial structures and its performance was the same when real experimental data were used. The artificial training structures contained valid organic molecules and they were optimized to ensure there were no too short intermolecular contacts or large voids present, but no intermolecular interactions were taken into account to make the training structures more chemically plausible. This would have required immense computational resources as each of the 48 million training structures would have been considered a separate case of the crystal structure prediction (CSP) problem. The usual CSP workflows include generating millions of different structures of the molecule in question and subsequently using ever more sophisticated energy calculations to identify the most plausible structure candidates (Beran, 2023 ▸).

Even excluding the CSP step, generation of the training examples required a considerable effort. Valid organic molecules represented as SMILES strings were obtained from the GDB-13 database (Blum & Reymond, 2009 ▸). Force-field calculations were performed to obtain a 3D molecular structure. The molecules were then placed into 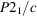 unit cells with random unit-cell parameters and the structures were further optimized to avoid atom clashes or large voids. This included shrinking or expanding the unit cells. Occasionally, additional metal atoms were placed in the unit cells. Eventually, 48 million structures were saved to be used for the neural network training.

It is fair to assume that training models capable of solving the phase problem for larger unit cells (say, up to 50 or even 100 Å) and in any symmetry (*P*1) will require even larger training data sets. This raises several questions regarding the training data generation, *e.g.* if any chosen finite set of organic molecules will be representative enough to lead to a generalized model and how to model different classes of compounds [inorganic, organic, coordination/metal–organic compounds, MOFs/COFs (metal–organic frameworks/covalent organic frameworks) *etc*.] in the training data.

In this article, we explore the possibility of simplifying artificial structure generation by not compromising the ability of the neural network to generalize the solution and be applicable to real experimental structures. The generation of artificial structures needs to be efficient, fast and preferably performed on-the-fly. The latter ensures flexibility in adapting the training data domain during neural network training and avoids transferring gigabytes or even terabytes of pre-saved data which is highly undesirable in deep learning projects. We demonstrate a strategy for training data generation that does not rely on chemically valid molecules, is fast and scalable. The PhAI neural network retrained on such data performs better than the original PhAI model when applied to experimental crystal structures with larger unit cells.

## 2. Methods

Routines for generating training structures were programmed in Python. Previously published PhAI neural network code (Larsen *et al.*, 2024 ▸) was used and adapted slightly to align it with the newly developed training data generation routines. Training was done on an Nvidia GeForce RTX 5090, GeForce RTX 4090 or A10G GPUs, using the AdamW optimizer with a batch size of 64, weight decay 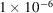 and initial learning rate 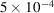. Learning rate was reduced manually on a loss plateau, first to 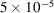 and then to 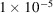. All models were trained on 100 million unique structures, except for retraining on the original PhAI data set (48 million structures, training for two epochs).

Diffraction data (amplitudes and ground-truth phases) were calculated on-the-fly during training. The original PhAI study sampled data resolution 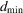 (1.0 to 2.0 Å) and completeness (85% to 100%) for each training structure. However, for the sake of simplicity, we chose a fixed resolution of 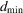 = 1.0 Å and 100% completeness.

A crucial part of PhAI architecture is phase recycling. Input for the neural network consists of reflection amplitudes and phases, which are initialized with random values and subsequently set to the model’s predictions from the previous cycle. A default value of three cycles was used for training. On inference the number of cycles was scanned from one to ten for each structure, initializing phases with either 0 or random values. The random phase initialization was done four times. Similarly to the original study, the results are reported for the most successful (least phase error) run for each structure. This is motivated by the fact that solutions to the phase problem are easily verifiable even without knowing the original structure, since wrong phase values lead to invalid or chemically improbable starting models.

Experimental crystal structures for testing and derivation of different structure parameter statistics were collected from the Crystallography Open Database (COD) (Vaitkus *et al.*, 2021 ▸) and the Cambridge Structural Database (CSD) (Groom *et al.*, 2016 ▸). For exploratory statistical studies, we used 1.25 million structures in the unit-cell dimension range 4 Å ≤ *a_n_* ≤ 50 Å and 38730 structures in the range 4 Å ≤ *a_n_* ≤ 10 Å. From the latter subset, 3086 structures in 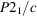 and equivalent settings were used for model evaluation. The testing results and trained models are available in the Zenodo archive (https://doi.org/10.5281/zenodo.17039016).

## 3. Results and discussion

### 3.1. General considerations

In deep learning, the training domain must contain samples as similar as possible to the ones for which the built model is intended to avoid a too large *synthetic-to-real domain gap* (Nikolenko, 2021 ▸). Only then can it be expected that the trained model will be general enough to perform well during inference. (In the context of machine learning, *inference* is the process of using a trained machine learning model to make predictions or generate outputs based on new, unseen data.) For solving the phase problem, one might wonder what this similarity of training and inference crystal structures means. Several parameters can be considered: unit-cell lengths and angles, unit-cell parameter ratio, unit-cell volume, density of the crystalline solid, element distribution, interatomic distances, to name a few.

We will formulate the phase problem in the context of deep learning as follows: the electron-density function (or electrostatic potential function in electron diffraction) 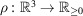 is a sum of 3D Gaussian-like functions. Its Fourier transform leads to a discrete complex function 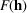 for which only the absolute values 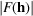 are known. The aim of a deep learning model is to recover the phase information 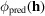, so that the inverse Fourier transform of 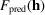 leads to ρ which is the said sum of 3D Gaussian-like functions.

This formulation suggests that it is not necessary for chemically valid molecular fragments to be present in the training structures. Generating a vast amount of training data that do not rely on valid molecules is computationally much more feasible. We further discuss measures to limit the scope of the vast training domain and design a targeted subspace aligned with the experimental crystal structures using data from databases.

### 3.2. Random sampling of unit-cell parameters

The most intuitive way for random structure generation would be randomly sampling unit-cell parameters (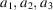) [or (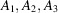) according to the probability theory notation] between some desired minimum and maximum values 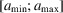 and then filling the unit cell with contents. We shall consider the effect of such a procedure on the distribution of the unit-cell volume. For the unit-cell parameters sampled from a uniform distribution, 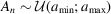, its probability density function (p.d.f.) is defined as
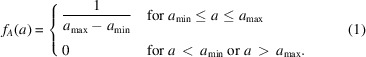
The volume of an orthorhombic unit cell is 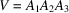. To derive the p.d.f. 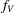 it is more convenient to reformulate the problem in terms of adding random variables rather than multiplying them, *i.e.*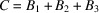, where 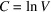 and 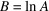. To find the p.d.f. of random variable *B*, we should first consider the integral of 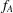, *i.e.* the cumulative distribution function (c.d.f.) 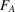: 
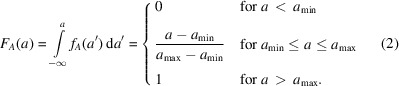
Since 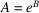, the c.d.f. of random variable *B* can be found: 
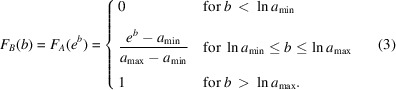
The p.d.f. of random variable *B* is the derivative of the corresponding c.d.f.: 
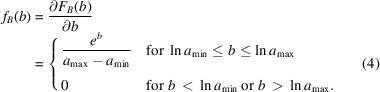
The p.d.f. of random variable *C* can then be obtained by the following convolution: 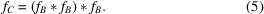
Finally, the p.d.f. of the unit-cell volume (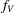) can be derived from 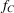 in a similar way as described for the transformation from 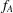 to 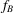.

**Figure 1:**
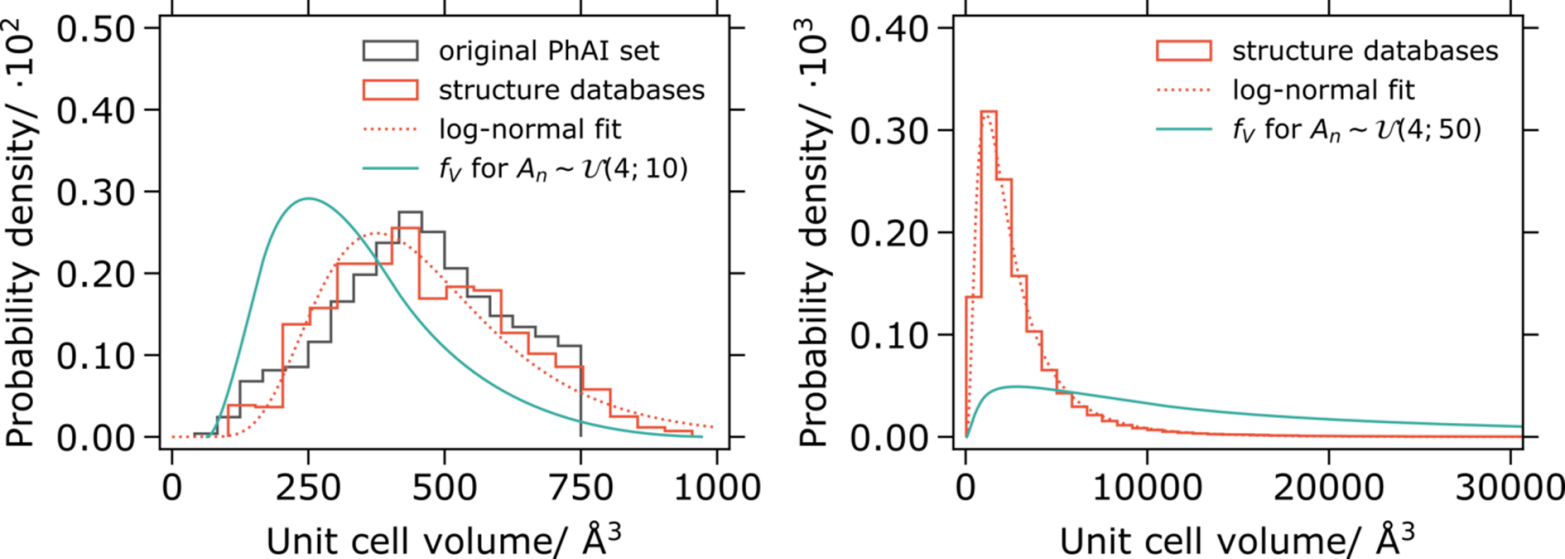
Left: distribution of the unit-cell volume for prospective artificial structures generated by sampling unit-cell dimensions uniformly between 4 and 10 Å; unit-cell volume distribution for 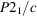 structures with 4 Å ≤ *A_n_* ≤ 10 Å found in databases with a fit to the log-normal distribution; unit-cell volume distribution of the original PhAI training data set. Right: distribution of the unit-cell volume for prospective artificial structures generated by sampling unit-cell dimensions uniformly between 4 and 50 Å; unit-cell volume distribution for all structures with 4 Å ≤ *A_n_* ≤ 50 Å found in databases with a fit to the log-normal distribution.

In Fig. 1 ▸ (left), the resulting distribution of the unit-cell volume (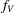) is given when 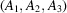 are sampled according to 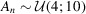. Also depicted are the distributions of the unit-cell volume for experimental crystal structures (in 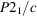 and with 4 Å ≤ *A_n_* ≤ 10 Å) and for the original PhAI training data set. The two latter distributions match very well. In Fig. 1 ▸ (right), the distribution of the unit-cell volume (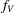) is given when 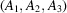 are sampled according to 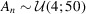. Furthermore, the data from the structure databases are presented for structures in any space group and with 4 Å ≤ *A_n_* ≤ 50 Å.

The results show that generating random structures by uniformly sampling unit-cell parameters will lead to a unit-cell volume distribution that matches the experimental structure set poorly. We will later show that such training data greatly hamper the ability of PhAI to generalize to structures far outside the training data domain. We further note that the unit-cell volume distribution of the experimental structures can be very well approximated with a log-normal distribution and we subsequently explore the possibility of generating the unit cells from sampled volumes.

### 3.3. Generating a unit cell from a sampled volume

To overcome the demonstrated discrepancy in unit-cell volume, we propose directly sampling *V* instead of 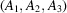. For example, the unit-cell volume can be sampled uniformly, 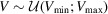 or according to the distribution found for experimental crystal structures, *e.g.*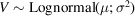. To obtain the unit-cell parameters (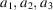) for a randomly sampled volume *V* and keep the cell lengths between some bounds 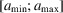, the following procedure is proposed.

**Figure 2:**
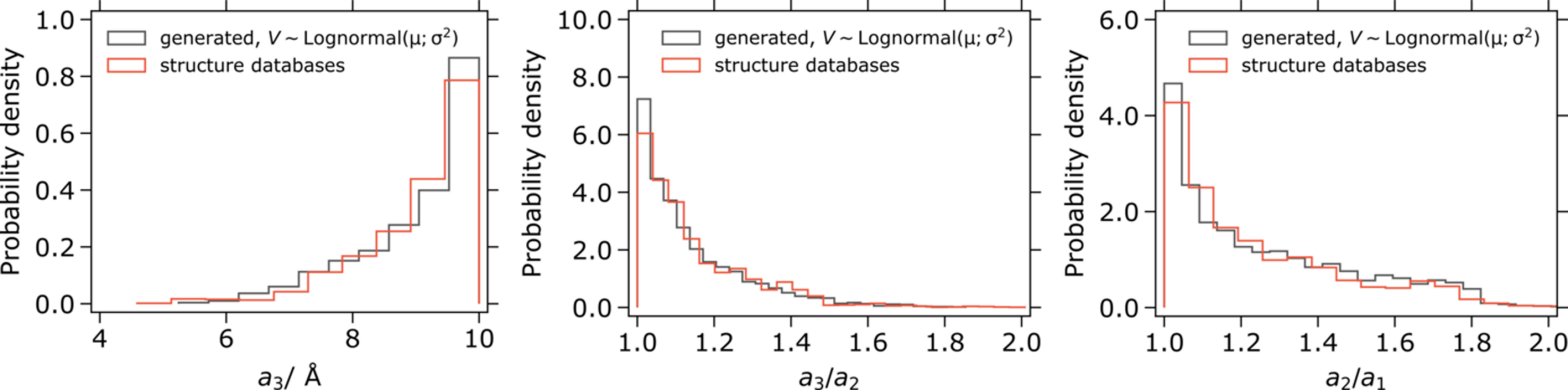
Left: distributions of the maximal unit-cell parameter for 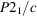 structures (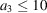) found in databases (*N* = 3086) and randomly sampled unit cells by our method. Middle, right: distributions of 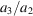 and 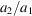 for the same database structures and unit cells sampled by our method. The distribution of *V* for our method was the log-normal fit of the corresponding distribution for the experimental crystal structures.

Starting with an orthorhombic unit cell and assuming unit-cell parameters are given in ascending order (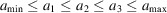), it is apparent that the largest possible value for 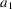 is reached when 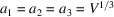. Therefore, the upper bound for 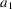 is 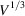:
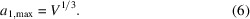
Furthermore, if 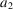 = 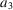 = 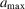, then 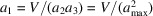, which gives a lower bound. In addition, we introduce a constant parameter *r* which represents a maximum ratio of two unit-cell parameters (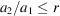, 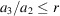) in order to avoid very unlikely large aspect ratios. From this restriction we derive another two lower bounds on 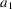. First: 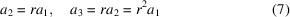

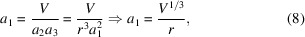
and the second to ensure that 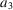 will not exceed 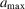: 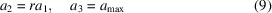

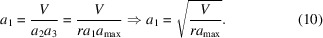
Thus, the final lower bound is 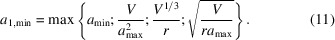
At this point 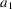 can be sampled from 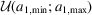. A similar process gives bounds for 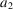: 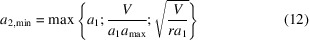

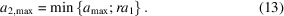
The third unit-cell parameter 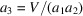 is then guaranteed to satisfy all given conditions. We observe in the structure databases that the unit-cell lengths are not independent and the respective ratios (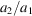 and 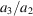) follow the exponential distribution. We chose the value of 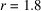 accordingly. The unit cells sampled by our method correspond well to the unit cells of experimental structures found in databases (Fig. 2 ▸).

After sampling, the values of 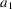, 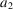, 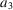 can be permuted randomly so as not to introduce any bias by ordering of cell lengths.

### 3.4. Generating oblique unit cells

**Figure 3:**
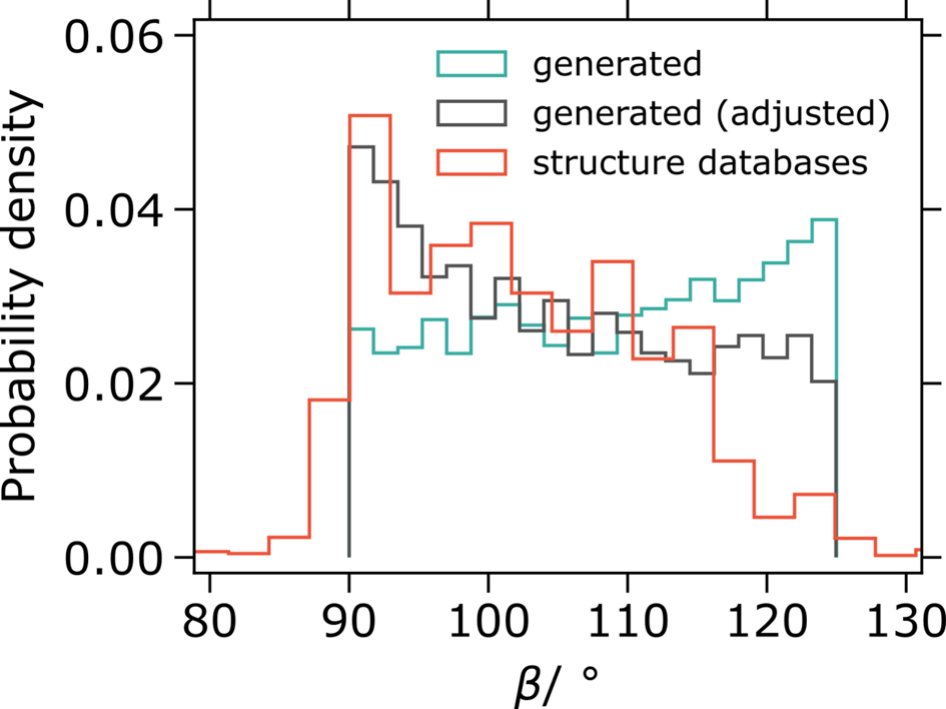
Distributions of the monoclinic angle of the experimental structures and the sampled values with and without an additional lower bound on 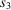.

The following skew matrix can be used to linearly transform an orthorhombic unit cell in order to obtain a monoclinic (*b*-unique) unit cell: 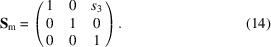
The value 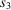 can be sampled in the range of 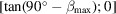. Thus, the monoclinic angle β will be in the range 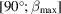. The new unit-cell vectors 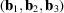 can be obtained from the orthogonal vectors 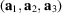 as 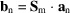. Since 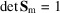 necessarily, the volume of the new unit cell will remain the same, whereas the cell lengths 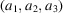 will change to 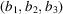. If it is necessary to keep the new cell lengths within the set bounds 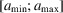, 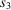 can be sampled in the following range: 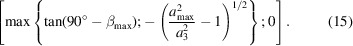
The sampled value of 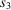 is related to the final monoclinic angle through a tangent function. Sampling 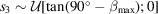 with 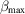 = 125° leads to the monoclinic angle distribution being shifted to higher angle values (Fig. 3 ▸, ‘generated’). That is not the case for experimental crystal structures.

By including the term 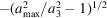 for the lower bound sampling, the resulting distribution [Fig. 3 ▸, ‘generated (adjusted)’] matches considerably better the experimental structures. Alternatively, 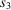 can be sampled, for example, exponentially to get even better correspondence to the distribution of the monoclinic angle found in experimental crystal structures.

If it is desirable to keep the new cell lengths identical to those of the orthogonal cell, *i.e.*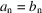, the new unit-cell vectors can be obtained as follows: 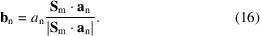
In this case, the volume of the unit cell will change when making it oblique, but the cell lengths will be kept the same. Finally, a similar transformation matrix can be used to obtain a triclinic unit cell: 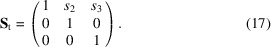


### 3.5. Filling the unit cell with atoms

The simplest approach to generating a crystal structure is to place all atoms randomly and uniformly within the unit cell. However, completely independent sampling of coordinates would lead to significant overlap between atoms. The restriction that atoms must be separated by at least a fixed distance 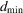 can be realized by rejection sampling, as in the following procedure:

(1) Choose the element to be placed. We draw 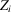 from an empirical distribution given by element frequencies in crystal structure databases (see Table S1 in the supporting information).

(2) Sample fractional coordinates 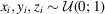.

(3) Check the distance between the sampled atom and all atoms already in the structure, considering also neighboring unit cells. If any distance is less than 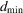, return to step (2) and generate new coordinates. If suitable coordinates are not found in ten iterations, discard the atom and move to the next.

(4) Repeat the steps 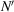 times, where 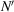 is the desired number of atoms in the asymmetric unit. The above limit of ten iterations per atom may result in less than 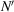 atoms being placed, but we find that this rarely occurs if a reasonable range of 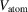 (the average available volume of an atom in the unit cell) is chosen.

We suspect that deep learning might benefit from training data that more closely match expected real-world data and propose another method for artificial structure generation. There are two main aspects in which structures generated by the above procedure differ from experimental crystal structures. First, randomly sampled coordinates always describe a general position, whereas in experimental structures atoms may also occur in special positions. Second, atoms are usually not distributed uniformly throughout the unit cell, but instead form clusters as molecules and polyatomic ions.

To address the first point, we propose populating special positions before placing any other atoms. In the space group 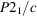, there are four distinct special positions corresponding to centers of inversion. By choosing *p* = 0.05 as the independent probability of any special position being occupied by an atom, we obtain 81.4% of generated structures with all atoms in general positions, 17.2% with one special position occupied and 1.4% with more than one special position occupied. These figures are similar to those observed in crystal structure databases (73.5%, 20.1% and 6.4% for 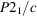 structures with *a_n_* ≤ 10 Å, respectively).

The second point requires a different approach to sampling atomic coordinates. We propose a procedure for generating ‘artificial molecules’ as follows:

(1) Independently sample occupancy (0 or 1) for each special position in the given space group, as described above.

(2) After populating special positions, place one atom *Z* in a general position with random coordinates.

(3) Choose the next element 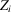 to be placed.

(4) Randomly select an atom *A* from the atoms already present in the structure. Sample the bond length 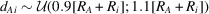, where 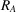 and 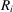 are covalent radii of atom *A* and of element 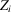, respectively. [The covalent radii are reported, for example, by Cordero *et al.* (2008 ▸).]

(5) Place atom *i* at a point sampled uniformly on a sphere with center *A* and radius 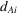 (Muller, 1959 ▸).

(6) For each atom *j* in the structure, check that 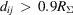 and 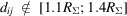, where 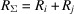. (In other words, two atoms must either form a covalent bond or be separated by at least 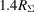. While non-covalent close contacts do occur in experimental crystal structures, they are rare in proportion to the total number of interatomic distances.) If any condition is violated, return to step (4). If suitable coordinates are not found in 20 iterations, return to step (3) and try again with a different element. If the search still fails, discard the atom and move to the next.

(7) Repeat steps (3)–(6) 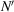 times, where 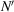 is the desired number of atoms in the asymmetric unit.

The distribution of element frequencies can be partitioned between special and general positions. For instance, we found in the crystal structure databases that 89% of all non-hydrogen atoms in general positions are C, N or O, while for special positions this proportion is only 49%; special positions are more often occupied by transition metals, owing to their tendency to form complexes with inversion symmetry. Therefore, separate consideration of special positions also serves to increase training set diversity and ensure sufficient representation of inorganic and metal–organic/coordination compound structures. More detailed figures on element frequencies are given in Table S1.

**Figure 4:**
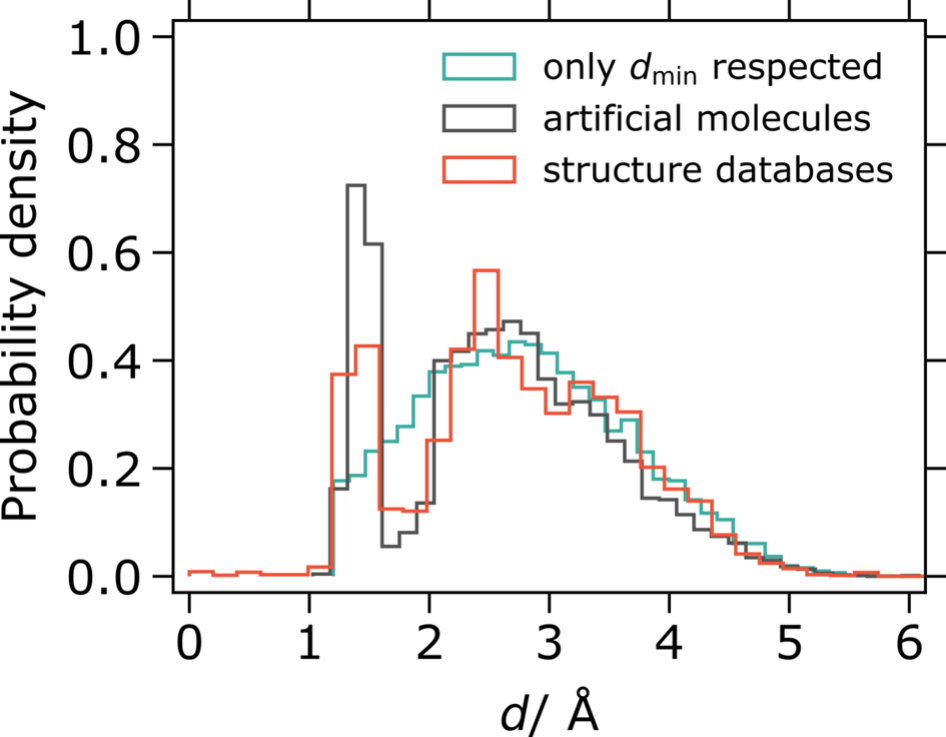
Distributions of interatomic distances in generated and experimental crystal structures. Two structure generation methods are given: placing atoms randomly but respecting a minimum interatomic distance 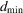 (here 1.2 Å); generating molecule-like clusters of atoms (artificial molecules).

The difference between the two approaches described here can most easily be seen when comparing distributions of interatomic distances (Fig. 4 ▸). For experimental crystal structures, we observe a peak corresponding to the length of a typical covalent bond, followed by a trough for distances between one and two bond lengths. Structures generated by the artificial molecule approach match this distribution closely. In contrast, the uniform atom approach leads to an inter­atomic distance distribution of significantly different shape. This is in effect the distribution of distances between random points in 3D space, truncated on the left at 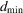 (Philip, 2007 ▸).

For both approaches we consider hydrogen and non-hydrogen atoms separately. Their number is determined by the average volume per non-hydrogen atom 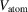, which we sample from 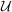
(7 Å^3^; 22 Å^3^) to cover the range of densities observed in crystal structure databases (Fig. S1 in the supporting information). The number of non-hydrogen atoms to place in the asymmetric unit 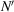 is then
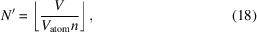
where *n* is the number of symmetry operators of the space group.

Afterwards the number of hydrogen atoms is determined by sampling the hydrogen mole fraction 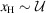
(0.3; 0.6): 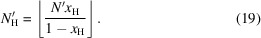
The database data show that the hydrogen-atom mole fraction in crystal structures is usually between 30 and 60%. Non-hydrogen atoms are always placed first in order to form a skeletal structure. In addition, we sample the isotropic atomic displacement parameter 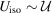
(0.01 Å^2^; 0.1 Å^2^) for each structure along with a deviation 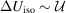
(−0.005 Å^2^; 0.005 Å^2^) for each individual atom. In Fig. S2, the distribution of the average 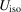 in experimental crystal structures is given. A few examples of artificial molecule structures are visualized in Fig. S3.

Both approaches of filling a random unit cell with contents are very fast. On a regular computer, a structure can be generated with an average time of 0.009 ms Å^−3^ with uniform atom distribution or 0.018 ms Å^−3^ with artificial molecules. For structures of dimension 4 Å ≤ *a_n_* ≤ 10 Å, the total average generation time is 6 ms (first approach) or 12 ms (second approach) per structure. This allows for training data generation on-the-fly during neural network training.

## 4. Retraining PhAI with artificial structure data

We retrained PhAI with training data generated according to three different unit-cell selection approaches (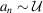, 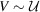 and 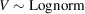) and two different atom placement approaches (random atom placement respecting 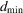 = 1.2 Å and generating artificial molecules as described above). For uniform atom placement, we also considered a training set of structures with all atoms being equal to assess the effect of different scatterers present in the structures. This is motivated by the fact that some ideas of direct methods are based on relationships only true for equal-atom structures.

The models were trained on 100 million structures each (for more details see the *Experimental* section). Then, each model was tested using 3086 experimental structures in 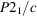 and with 4 Å ≤ *a_n_* ≤ 10 Å found in crystal structure databases. As in the original study of PhAI, we used the correlation coefficient *r* between the phased and the true electron-density map to assess the success of phasing. The results are summarized in Table 1 ▸ where the percentage of the solved experimental crystal structures, *f*, and the median values of *r* are listed. We consider a structure as solved if 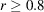.

The data in Table 1 ▸ show that all structure generation strategies except for equal-atom cases perform very well and are comparable with the PhAI model trained on the original training set. The percentage of solved structures is nearly 100%. In addition, the median *r* values indicate that the solutions are mostly very accurate, *i.e.* almost all predicted phases are correct.

A very different picture emerges when we test the trained PhAI models for solving 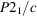 structures with larger unit cells, *i.e.* with *a_n_* < 20 Å but at least one of the lattice parameters larger than 10 Å to exclude the already tested small unit-cell structures. The data are summarized in Table 2 ▸.

We can conclude that the best performance of PhAI can be reached when the training data are composed of structures generated with artificial molecules and when the unit-cell volume is sampled instead of the lattice parameters. In this case, 86% of large unit-cell structures could be considered to have a good solution. Besides, the median value of *r* being 0.91 indicates that the density map accuracy is high. Models with randomly sampled atoms and uniformly sampled unit-cell parameters perform significantly worse. A particularly bad structure solving performance (52%) is for the model where the training structures are generated by randomly sampling unit-cell parameters and randomly placing atoms but respecting some 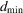. This could be considered one of the most intuitive scenarios for random structure generation.

**Figure 5:**
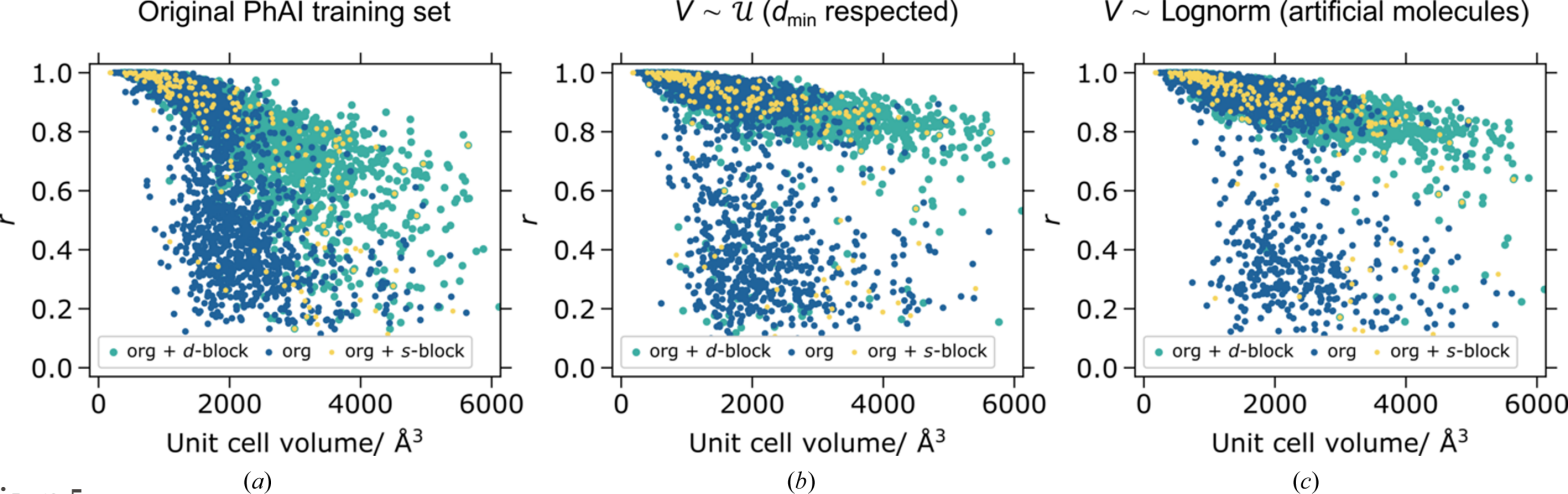
Correlation coefficient *r* versus unit-cell volume (*N* = 5000) for PhAI models trained: (*a*) on the original PhAI training set; (*b*) on structures generated by sampling the volume uniformly and randomly placing the atoms but respecting 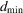; (*c*) on structures generated by sampling the volume log-normally and filling the unit cell with artificial molecules. The test structures are segregated by the compound classes as described in the text.

The tested large unit-cell data set (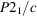, *a_n_* < 20 Å, *N* = 5000) can be segregated into three compound classes (organic molecules only; structures containing at least one *s*-block element; structures containing at least one *d*-block element). The corresponding *r* values for three of the models are summarized in Fig. 5 ▸. For other models, see Fig. S4.

A similar tendency can be observed in all three graphs in Fig. 5 ▸ – the structure solving performance starts to fail above the unit-cell volume of 1000 Å^3^. Indeed, in all cases the training data did not contain any structures with *V* > 1000 Å^3^. Furthermore, the limited input tensor of PhAI cannot accept reflections with 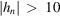. Nevertheless, there is a substantial improvement in phasing performance going from PhAI models trained on the original training set to 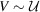 (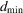 respected) to 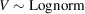 (artificial molecules) training sets. It appears that, similar to the conventional phasing methods, it is easier to solve crystal structures containing ‘heavy atoms’. Note that most of the failed cases (also in relative numbers) for the best PhAI model [Fig. 5 ▸(*c*)] are structures of purely organic molecules.

**Figure 6:**
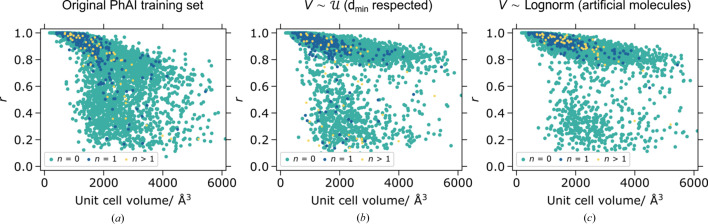
Correlation coefficient *r* versus unit-cell volume (*N* = 5000) for PhAI models trained: (*a*) on the original PhAI training set; (*b*) on structures generated by sampling the volume uniformly and randomly placing the atoms but respecting 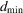; (*c*) on structures generated by sampling the volume log-normally and filling the unit cell with artificial molecules. The test structures are segregated by the number of special positions *n* occupied.

We further segregated the tested structures by the number of special positions *n* present (Fig. 6 ▸; Fig. S5 for all models). Here, we see that the best performing model (trained on 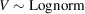 artificial molecule data) can deal with almost all tested experimental crystal structures for which there is at least one special position occupied. It is not true for the model trained on structures with randomly placed atoms, *i.e.* without special positions in the respective training set structures. In addition to more structures containing special positions being above the *r* = 0.8 threshold in Fig. 6 ▸(*c*), there is a systematic shift to better *r* values as compared with the results in Fig. 6 ▸(*b*).

We further notice in Fig. 6 ▸ (and Fig. S5) that two subsets of points emerge for the best phasing model, *i.e.* solutions with *r* of around 0.8 and above for nearly 90% of the structures and solutions with an *r* below 0.5. This shows that just changing the training data of the same neural network architecture can have a substantial influence on the generalization of the phase problem solution.

**Figure 7:**
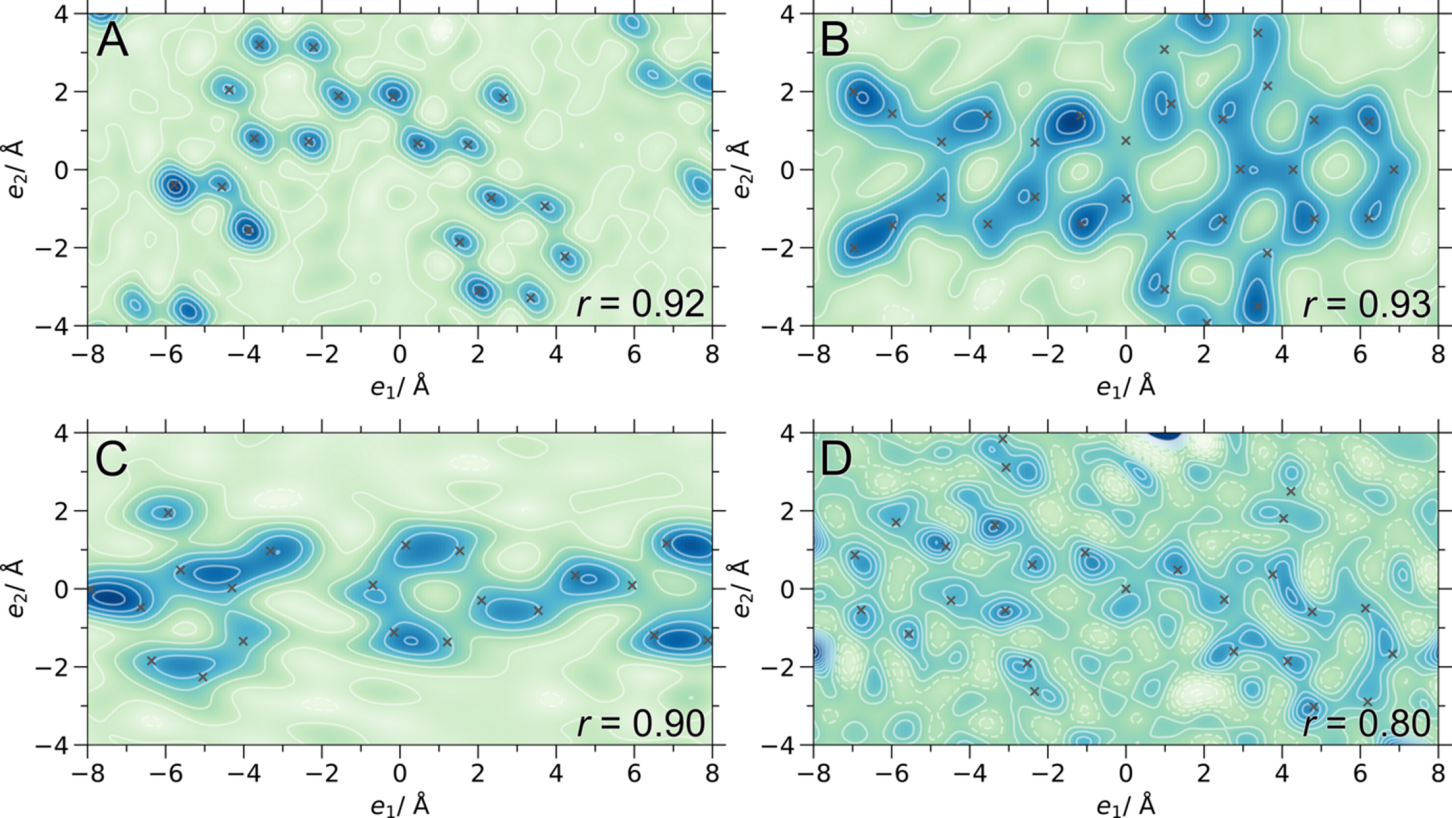
Density map projections (0.5 Å slabs in 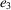 summed in 0.05 Å steps) of selected large unit-cell structures phased with the PhAI model trained on 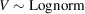 artificial molecule data. (*a*) CSD ARUZIY, COD 7225798 (Hall *et al.*, 2016 ▸), *V* = 1264 Å^3^. (*b*) CSD PIDDEP (Michalsky *et al.*, 2022 ▸), *V* = 2201 Å^3^. (*c*) CSD NUQHAK, COD 7222071 (Mir *et al.*, 2015 ▸), *V* = 3203 Å^3^. (*d*) CSD DYEETS (Kaneda *et al.*, 1977 ▸), *V* = 3068 Å^3^. True atomic positions are marked with crosses. Correlation coefficients *r* between the phased maps shown and true density maps are indicated.

In Fig. 7 ▸, phased electron-density map projections of selected large unit-cell structures comprising some reasonably flat molecules are given. The maps were phased with the PhAI model trained on 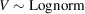 structures with artificial molecules. The maps are primarily distorted because of the limited data resolution in specific directions as the PhAI input tensor can only fit reflections with 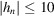. Despite these shortcomings, the maps are interpretable, but more importantly, they illustrate the ability of the used training data from one domain (small unit-cell structures with *V* < 1000 Å^3^) to generalize to unseen data comprising structures with larger unit cells.

## 5. Conclusions

We conclude that, for solving small unit-cell structures with the neural network PhAI, there is no significant difference regarding how the training data are generated, as long as no equal-atom structures are used. Very clear differences emerge when the ability of the neural network to generalize for larger unit-cell structures is tested. One of the proposed methods, *i.e.* sampling the unit-cell volume according to the log-normal distribution of the unit-cell volumes found for experimental structures and filling the unit cell with artificial molecules, leads to a more general training set. By resorting to structural databases and using chemical constraints in a statistical manner, it is possible to design synthetic training data resulting in a reduced synthetic-to-real domain gap. Moreover, there is a clear indication of a good generalization to unseen data that are significantly outside the used training data domain. An additional advantage of the proposed method is the ability to generate the data on-the-fly and dynamically choose the input parameters, like unit-cell volume range, space group and element distributions, to name a few.

## Supplementary Material

Additional statistical parameters on crystal structures, extended testing results. DOI: 10.1107/S2053273325009428/tw5015sup1.pdf

## Figures and Tables

**Table 1:** Median values of *r* and fraction of solved experimental crystal structures (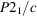, *a_n_* ≤ 10 Å, *N* = 3086) with the PhAI neural network retrained with different training data generation strategies

	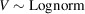 (6.04; 0.394)		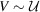 (160 Å^3^; 1000 Å^3^)		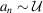 (4 Å; 10 Å)	
	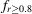	med(*r*)	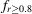	med(*r*)	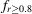	med(*r*)
Artificial molecules	99.8%	0.999996	99.6%	0.999996	99.8%	0.999993
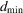 respected	99.6%	0.999987	99.2%	0.999989	99.3%	0.999988
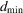 respected (equal atoms)	81.6%	0.96	72.5%	0.95	79.7%	0.96
						
Original PhAI training set	99.9%	0.999997				

**Table 2:** Median values of *r* and fraction of solved experimental crystal structures (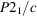, *a_n_* < 20 Å, *N* = 5000) with the PhAI neural network retrained with different training data generation strategies

	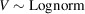 (6.04; 0.394)		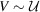 (160 Å^3^; 1000 Å^3^)		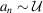 (4 Å; 10 Å)	
	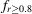	med(*r*)	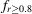	med(*r*)	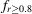	med(*r*)
Artificial molecules	86%	0.91	85%	0.91	78%	0.89
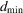 respected	79%	0.90	82%	0.91	52%	0.81
						
Original PhAI training set	60%	0.86				

## Data Availability

PhAI models retrained with different data are accessible from https://doi.org/10.5281/zenodo.17039016.
