# Supplementary material for: On artificial crystal structure generation for solving the phase problem with deep learning
Source: Acta Crystallogr A Found Adv. 2026 Jan 1;82(Pt 1):32–40. doi: 10.1107/S2053273325009428 (PMC12785641; doi:10.1107/S2053273325009428)
Supplement: Supplementary file 1 [file a-82-00032-sup1.pdf]

FOUNDATIONS
ADVANCES

Volume 82 (2026)

Supporting information for article:

On artificial crystal structure generation for solving the phase problem with deep learning

Džonatans Miks Melgalvis and Toms Rekis

Contents

1 Element frequencies in experimental crystal structures 2

2 Additional statistics on crystal structure parameters 5

3 Examples of artificial crystal structures 6

4 Phasing results of PhAI neural network trained on different data 7

5 Running the retrained PhAI models 10

1 Element frequencies in experimental crystal structures

Z	Element symbol	All positions	General positions	Special positions
2	He	—	—	—
3	Li	5	4	50
4	Be	—	—	10
5	B	52	50	120
6	C	6932	7010	2460
7	N	702	701	730
8	O	1220	1210	1750
9	F	306	308	200
10	Ne	—	—	—
11	Na	8	6	120
12	Mg	4	2	120
13	Al	9	7	130
14	Si	36	35	130
15	P	84	83	180
16	S	120	119	190
17	Cl	150	147	310
18	Ar	—	—	—
19	K	7	6	100
20	Ca	3	2	80
21	Sc	1	1	20
22	Ti	5	4	60
23	V	5	5	40
24	Cr	4	3	40
25	Mn	10	8	150
26	Fe	23	19	270
27	Co	15	12	200
28	Ni	15	11	210
29	Cu	30	25	330
30	Zn	15	12	180
31	Ga	3	2	50
32	Ge	4	3	50
33	As	4	4	30
34	Se	10	9	50
35	Br	31	30	90
36	Kr	—	—	—
37	Rb	1	1	20

Z	Element symbol	All positions	General positions	Special positions
38	Sr	1	1	30
39	Y	2	1	20
40	Zr	3	2	40
41	Nb	2	2	30
42	Mo	15	14	50
43	Tc	—	—	—
44	Ru	11	11	40
45	Rh	6	5	30
46	Pd	9	8	70
47	Ag	13	12	80
48	Cd	6	5	100
49	In	2	1	40
50	Sn	6	5	60
51	Sb	5	4	50
52	Te	3	2	30
53	I	22	20	110
54	Xe	—	—	—
55	Cs	2	1	30
56	Ba	2	1	50
57	La	2	1	30
58	Ce	1	1	30
59	Pr	1	1	10
60	Nd	1	1	20
61	Pm	—	—	—
62	Sm	1	1	20
63	Eu	2	2	20
64	Gd	2	2	20
65	Tb	2	1	20
66	Dy	2	2	20
67	Ho	1	1	10
68	Er	1	1	20
69	Tm	—	—	10
70	Yb	1	1	20
71	Lu	1	1	10
72	Hf	1	—	10
73	Ta	1	1	20
74	W	17	17	40
75	Re	4	4	20

Z	Element symbol	All positions	General positions	Special positions
76	Os	3	3	10
77	Ir	4	4	20
78	Pt	7	6	60
79	Au	8	7	50
80	Hg	2	2	20
81	Tl	1	—	10
82	Pb	3	2	60
83	Bi	3	2	40
84	Po	—	—	—
85	At	—	—	—
86	Rn	—	—	—
87	Fr	—	—	—
88	Ra	—	—	—
89	Ac	—	—	—
90	Th	1	—	10
91	Pa	—	—	—
92	U	3	2	40

Table S1: Frequencies of elements found in crystal structure databases, represented as number of occurrences per 10000 non-hydrogen atoms. For special positions, values are rounded to nearest 10 because of significantly smaller sample size. — signifies less than 1 occurrence per 10000.

2 Additional statistics on crystal structure parameters

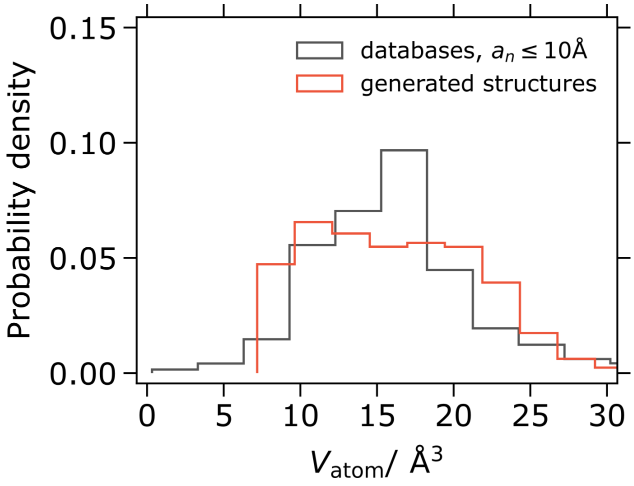

Figure S1: Distribution of V_{atom} for the experimental structures and generated structures.

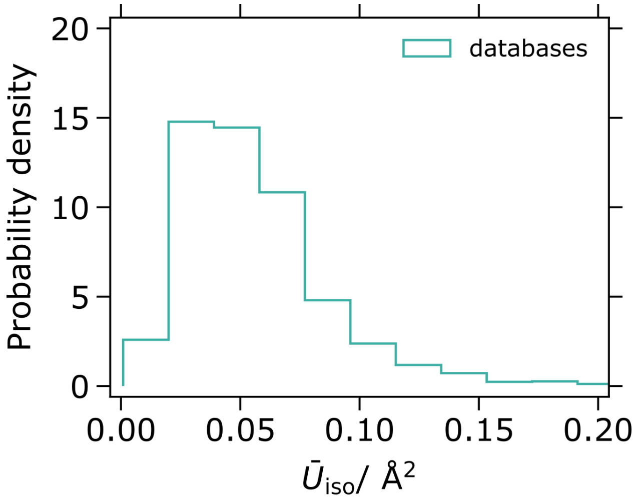

Figure S2: Distribution of the average U_{iso} for the experimental structures.

3 Examples of artificial crystal structures

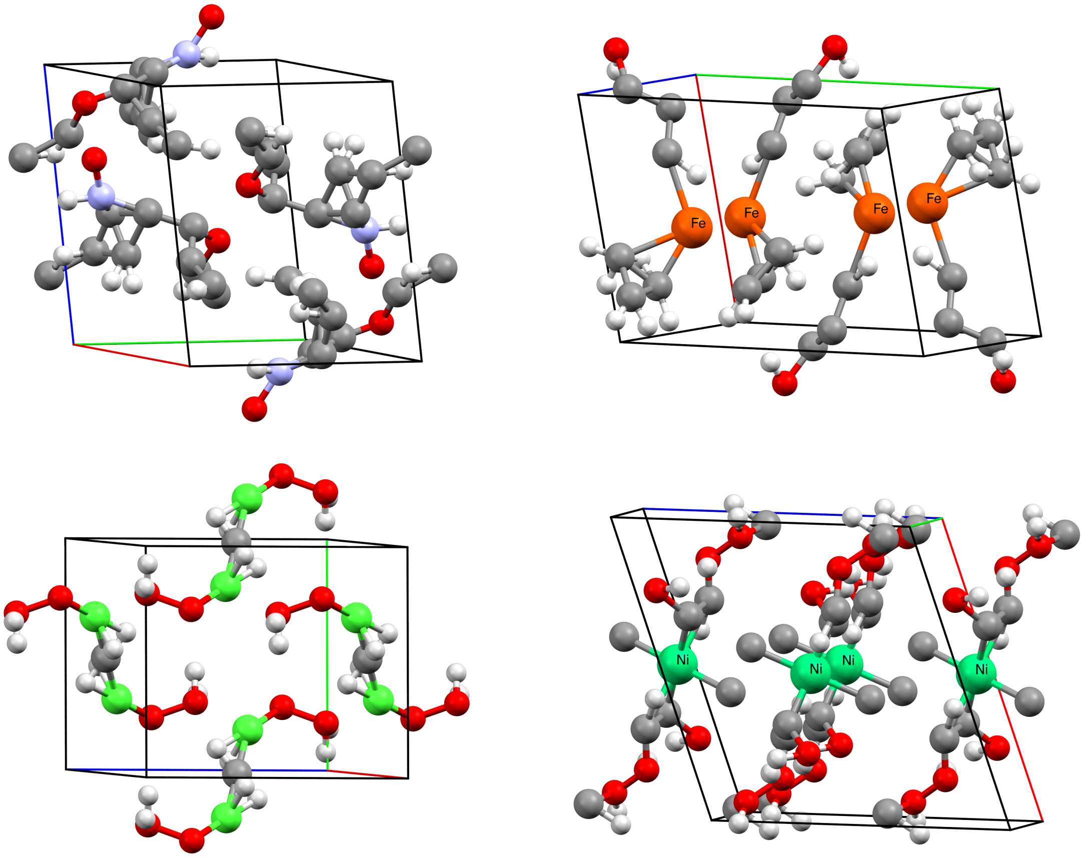

Figure S3: Example structures generated by the artificial molecule approach in space group $P2_1/c$. Top left: organic structure (only C, N, O, H atoms). Top right: structure with metal atom in a general position. Bottom left: structure with C atom in a special position. Bottom right: structure with metal atom in a special position.

4 Phasing results of PhAI neural network trained on different data

The phasing results for all models referencing all tested structures with their COD and/or CSD refcodes are available in files `testing_small.csv` and `testing_large.csv`. The model numbering is as follows:

- `model_27`: PhAI original training set
- `model_34`: $V \sim$ Lognorm, artificial molecules
- `model_35`: $V \sim \mathcal{U}$, artificial molecules
- `model_36`: $a_n \sim \mathcal{U}$, artificial molecules
- `model_37`: $V \sim \mathcal{U}$, random atoms but respecting d_{min}
- `model_38`: $V \sim$ Lognorm, random atoms but respecting d_{min}
- `model_39`: $V \sim \mathcal{U}$, random equal atoms but respecting d_{min}
- `model_40`: $V \sim$ Lognorm, random equal atoms but respecting d_{min}
- `model_41`: $a_n \sim \mathcal{U}$, random atoms but respecting d_{min}
- `model_42`: $a_n \sim \mathcal{U}$, random equal atoms but respecting d_{min}

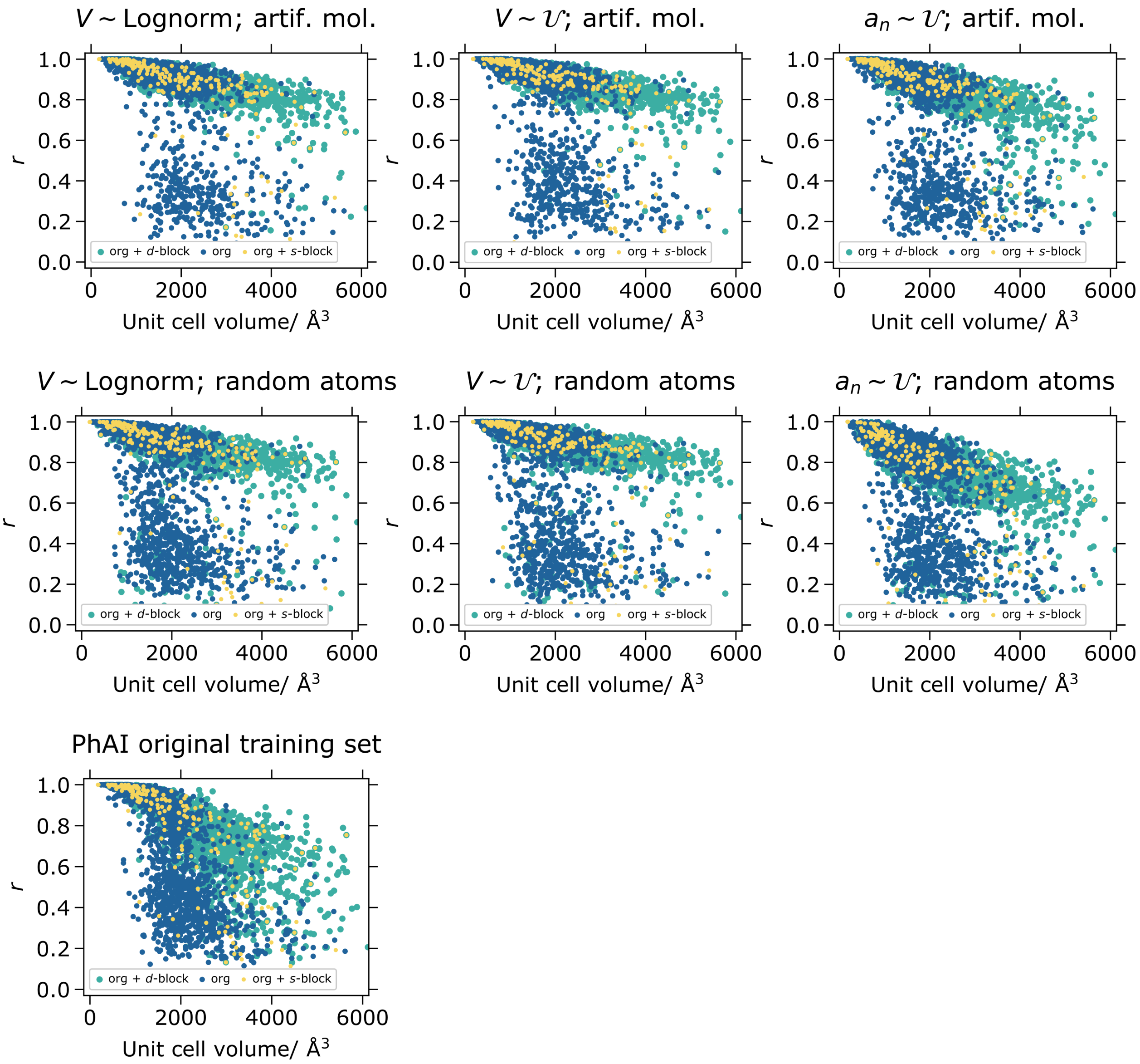

Figure S4: Correlation coefficient r versus unit cell volume for PhAI models trained on different training data. The experimental test structures ($P2_1/c$, $a_n < 20\,\text{Å}$, $N = 5000$) are segregated by the compound classes as described in the text.

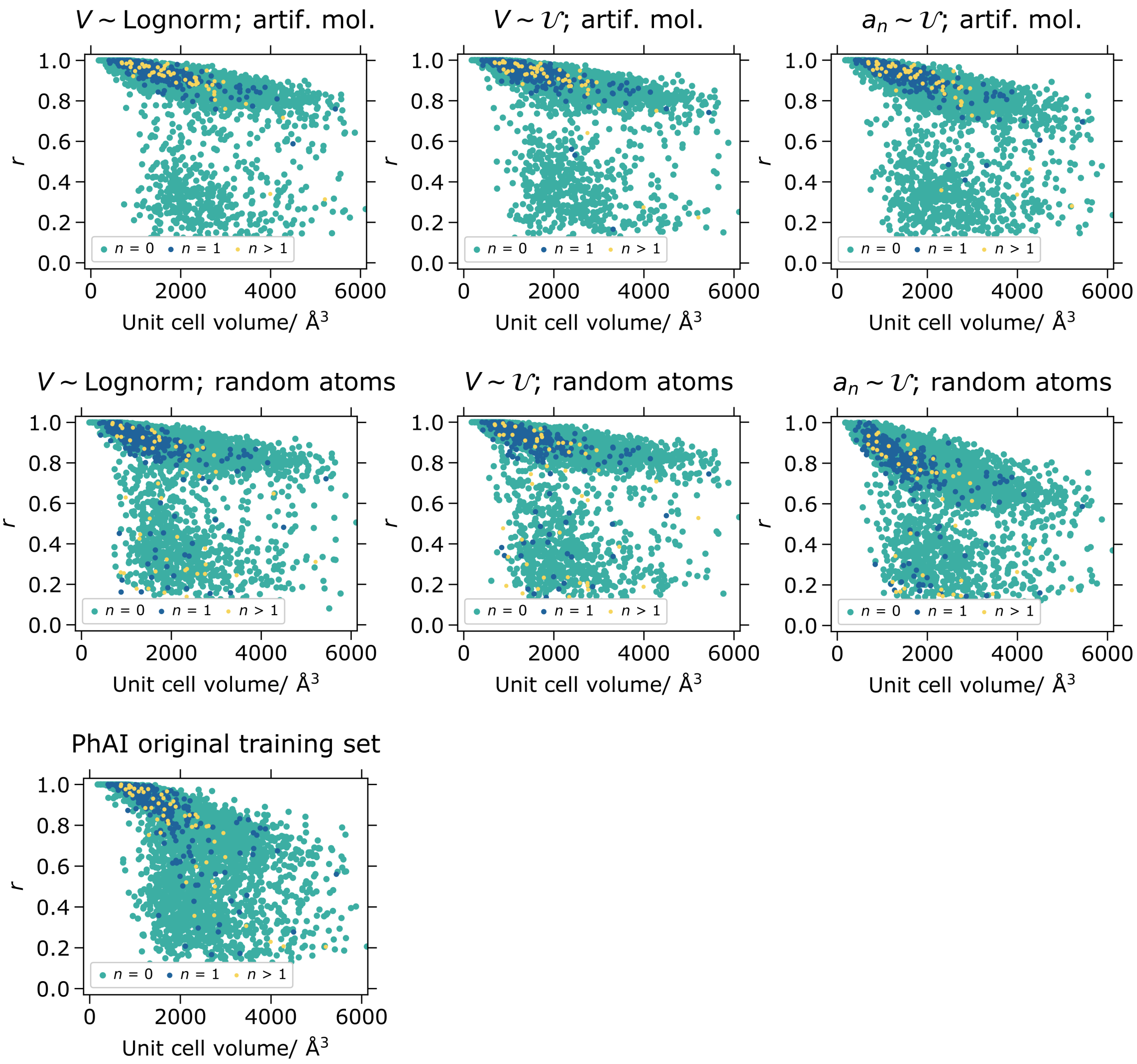

Figure S5: Correlation coefficient r versus unit cell volume for PhAI models trained on different training data. The experimental test structures ($P2_1/c$, $a_n < 20\,\text{Å}$, $N = 5000$) are segregated by the number of special positions n occupied.

5 Running the retrained PhAI models

An adapted version of the wrapper script for PhAI, which includes models retrained with various training data generation strategies can be accessed here: 10.5281/zenodo.17039016.

The original script was published in: Larsen, A. S., Rekis, T. & Madsen, A. (2024). *Science*, 385(6708), 522–528.

Python dependencies: numpy, pandas, torch, einops.

Usage: python PhAI_adapted.py -i <inputfile> [-m <model> -n <n> -p <p> -t]

-i <inputfile> is a plain text .hkl file containing a list of Miller indices and the absolute values of the structure factors. The list of reflections will be reindexed according to the conventions used for PhAI, structure factor absolute values will be scaled so that the strongest reflection has a value of 1.0. In case symmetrically equivalent reflections are present, a simple averaging will be performed.

Output will contain signed structure factors for reflections with $-10 < h < 10$, $0 \leq k < 10$, $0 \leq l < 10$ (after reindexing). Phase extension is not supported in this version, only phases for the reflections given in input will be produced.

-m <model> is the name of the model to use for phasing (see paper for details). Available values:

- mols_V-lognorm: trained with artificial molecules and $V \sim \text{Lognorm}(6.04; 0.394)$ [default]
- mols_V-unif: artificial molecules, $V \sim \mathcal{U}(160; 1000)$
- mols_a-unif: artificial molecules, $a_n \sim \mathcal{U}(4; 10)$
- rand_V-lognorm: trained with randomly distributed atoms, $V \sim \text{Lognorm}(6.04; 0.394)$
- rand_V-unif: randomly distributed atoms, $V \sim \mathcal{U}(160; 1000)$
- rand_a-unif: randomly distributed atoms, $a_n \sim \mathcal{U}(4; 10)$
- equal_V-lognorm: trained with randomly distributed equal atoms, $V \sim \text{Lognorm}(6.04; 0.394)$
- equal_V-unif: randomly distributed equal atoms, $V \sim \mathcal{U}(160; 1000)$
- equal_a-unif: randomly distributed equal atoms, $a_n \sim \mathcal{U}(4; 10)$
- PhAI_retrained: trained with the original PhAI dataset

-n <n> is the number of phasing cycles with a default value of 10, which means that the neural network's prediction of phases will be used as the new input for 9 (n-1) consecutive cycles. Setting -n 1 gives a single prediction without phase recycling. Using at least 5 cycles is recommended, as recycling greatly improves phasing accuracy.

`-p <p>` is either 0 or 1. The default value is 1, which means that the initial phases used in the first execution of the neural network will be set randomly. In case the phasing run gives unsatisfactory results, it is recommended to try again; a different set of random initial phases will be generated and the result may improve. Setting p to 0, the initial phases will all be 0 rad (deterministic run).

`-t` is an option for exporting phasing trajectories, i.e. when present a file of the solution provided by PhAI will be saved after each phasing cycle. In case phasing gives unsatisfactory results, these files can be examined, as in rare cases instability occurs and intermediate cycles give better results than the final cycle.

Example: `python PhAI_adapted.py -i COD_7225798.hkl -m mols_V-lognorm -n 10 -p 1 -t`

Folowing output will be exported:

- COD_7225798_1.F (first prediction starting with random phases (p=1) containing all reflections with $|h_n| < 10$ found in COD_7225798.hkl)
- COD_7225798_2.F (second prediction using phases from the first prediction as input)
- ...
- COD_7225798.F (final prediction)

Note that the given example (COD 7225798) is reported in $P2_1/n$ in COD. The .hkl file has been reindexed according to $P2_1/c$.
